# Integrative neural mechanisms for social communication of learned vocal behavior

**DOI:** 10.3389/fnint.2025.1650323

**Published:** 2025-10-17

**Authors:** Katherine L. Anderson, Osceola Whitney

**Affiliations:** ^1^Department of Biology, The City College of New York, City University of New York, New York, NY, United States; ^2^Biology PhD Program, The Graduate Center, City University of New York, New York, NY, United States; ^3^CUNY School of Medicine, City University of New York, New York, NY, United States

**Keywords:** songbird, zebra finch, social behavior, systems neuroscience, dopamine, oxytocin

## Abstract

Context-sensitive behaviors are crucial for the adaptive success of many organisms. Investigating neural processes that facilitate context-sensitive behavior requires knowledge of the molecular signaling and anatomical brain connectivity within and between relevant brain networks. Here, we outline the roles of oxytocin and dopamine signaling systems in context-sensitive singing in songbirds. Additionally, using the recently compiled songbird connectome, we review anatomical connectivity between vocal-motor and social brain networks that may facilitate context-sensitive singing. We present a model for context-sensitive adaptability of singing behavior in songbirds. We propose that the medial preoptic nucleus of the hypothalamus may serve as the output nucleus of the social behavior network, influencing oxytocin-mediated dopamine delivery to the vocal control network, in a context-sensitive manner. As many components of this model are conserved across species, we speculate that this proposed model can be generalized to facilitate context-sensitive motor behaviors across vertebrate species. Overall, we emphasize the importance of investigating each component of our proposed model, within a single species. This perspective aims to uncover how integrated neural mechanisms give rise to behavior.

## 1 Introduction

### 1.1 Context-sensitive singing behavior in a songbird

Oscine songbirds are a unique model system for studying context-sensitive modifications to learned behavior. Over half of all avian species, which includes oscine songbirds, but also parrots and hummingbirds, exhibit vocal learning, a rare trait among mammals. One avian species in particular, the oscine zebra finch *(Taeniopygia guttata)* modifies the timing, sequence stereotypy, and frequency stereotypy of their learned song when directing it toward a potential mate, compared to songs not directed at an identified individual (Sossinka and Böhner, 1980; Zann, 1996). Presentation of a female to juvenile male zebra finches can also elicit this context-sensitive song phenotype, prior to the end of the song-learning developmental phase (Kojima and Doupe, 2011). These context-specific song alterations are extremely salient for females of this species, who spend more time in proximity of speakers delivering audio playbacks of female-directed singing than undirected singing (Woolley and Doupe, 2008), making context-sensitive song production advantageous for mate selection. In the following review, we explore the contributions of two neural networks (i.e., vocal control network, social behavior network) and two neurotransmitters (i.e., dopamine, oxytocin) in the production of context-sensitive song. We primarily review data reported in zebra finches, as context-sensitive vocal-motor behavior in this species is extremely well-studied, but include findings from other songbirds. Additionally, we provide insight into the neural pathways underlying behavior in non-avian model organisms that exhibit context-sensitive motor or vocal-motor behaviors.

### 1.2 Dual control through vocal control and social behavior networks

**Figure 1:**
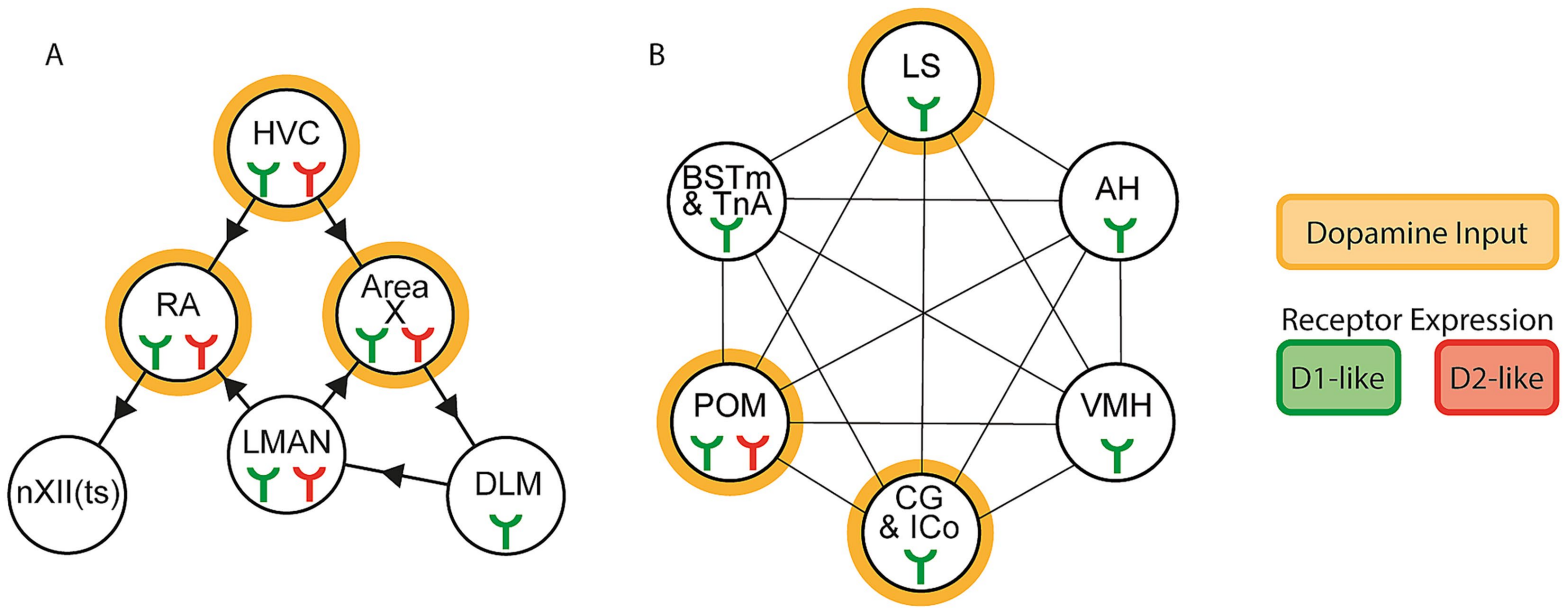
Dopamine signaling across the vocal control and social behavior networks. Expression of D1-like (green icon) and D2-like (red icon) dopamine receptors within the **(A)** vocal control network and **(B)** social behavior network. Innervation by dopamine synthesizing regions, or local synthesis of dopamine, is represented by an orange ring around the node. Arrowheads between nodes indicate anatomical connectivity, connecting lines without arrowheads indicate reciprocal connections.

In songbirds, learning, production, and context-sensitive modification of vocalizations are each reliant on a network of nucleated brain regions that are specialized for vocal-control (Nottebohm et al., 1976), analogues of which exist in humans (Jarvis et al., 2005; Moorman et al., 2012). This “vocal control network” can be split into two smaller pathways, one specialized for motor production of learned vocalizations and a second specialized for vocal plasticity. The posterior motor pathway includes a motor nucleus HVC (proper name), the robust nucleus of the arcopallium (RA), and the tracheosyringeal subdivision of the hypoglossal nucleus (nXII[ts]), a brainstem nucleus that innervates vocal muscles. In this motor pathway, HVC serves as the pattern generator for song such that manipulations of neural spiking pattens in HVC neurons directly correlate to alterations of resulting song (Long and Fee, 2008). The anterior forebrain pathway includes a striatal region Area X (proper name), the lateral magnocellular nucleus of the nidopallium (LMAN), and the dorsolateral nucleus of the medial thalamus (DLM), creating a cortical-basal ganglia loop, necessary for song plasticity during development and generation of adaptive modifications (Bottjer et al., 1984; Brainard and Doupe, 2000; Kao et al., 2005; Ölveczky et al., 2005). These two sub-pathways are anatomically connected via two projections from HVC to Area X and from LMAN to RA (as depicted in Figure 1A). LMAN inhibits context-sensitive modifications to fundamental frequency variation in song (Kao and Brainard, 2006). Additionally, pharmacological inactivation of LMAN during a learned syllable pitch-shift paradigm caused regression in the learned change to pitch (Andalman and Fee, 2009). These data suggest that the anterior forebrain pathway contributes to variation in song frequency, potentially utilizing the anatomical connection from LMAN to RA.

Facilitating context-sensitive singing, the vocal control network exhibits context-sensitive neural spiking and context-sensitive neural activity-dependent gene expression (Jarvis et al., 1998; Hessler and Doupe, 1999; Kao et al., 2008). Such dynamic context-sensitive neural activity patterns may influence downstream molecular processes, such as gene translation (Whitney and Johnson, 2005). Early efforts to explore the songbird singing transcriptome via the creation of whole brain or telencephalon cDNA libraries examined undirected and female-directed singing adult male zebra finches (Jarvis et al., 2002; Wada et al., 2006). Unfortunately, more recent studies utilizing RNA-sequencing in zebra finches and other songbird species have primarily focused on one singing condition or have excluded singing status from their analyses altogether (Drnevich et al., 2012; Whitney et al., 2014; Burkett et al., 2018; Ko et al., 2021). In the vocal control network, mRNA expression of 10% of transcribed genes in the songbird genome are induced by singing in isolation (Whitney et al., 2014). Many of these song-regulated genes are uniquely transcribed in one of the four cortical nodes of the vocal control network (HVC, RA, Area X, or LMAN) (Whitney et al., 2014). There is a significant lack of high-resolution transcriptomic analysis of social influence on the singing transcriptome in any songbird species. Nonetheless, the general mechanisms by which the isolated vocal control network manages context-sensitive song production are well-studied, but the integration of other defined neural systems (i.e., the social behavior network) that likely drive context-sensitive changes to behavior are less understood.

Studies in songbirds suggest that a social behavior network is involved in context-sensitive behavior. The social behavior network exhibits context-sensitive activation during singing (Heimovics and Riters, 2007; Anderson et al., 2023), alluding to a functional role for this network in context-sensitive singing. Evidence suggests that this social behavior network is fully interconnected and conserved across vertebrates (Newman, 1999; Goodson, 2005). In songbirds, the following brain regions compose six nodes of this social behavior network: the anterior hypothalamus (AH), medial bed nucleus of the stria terminalis (BSTM) and nucleus taenia (TnA), central gray (CG) and intercollicular nucleus (ICo), lateral septum (LS), medial preoptic nucleus of the hypothalamus (POM), and the ventromedial nucleus of the hypothalamus (VMH). In this proposed social network, the songbird TnA is considered homologous to the mammalian medial amygdala (Goodson, 2005). Additionally, considering similarities of the distribution of several neuropeptides across the songbird CG and ICo and the mammalian periaqueductal gray (PAG) (Kingsbury et al., 2011; Goodson and Kingsbury, 2013), we consider CG and ICo to serve together as the node of the social behavior network homologous to mammalian PAG. Investigations of the songbird social behavior network have highlighted its functional role in innate social behaviors, like proximity to adult conspecifics (Goodson et al., 2005), nest building behaviors (Edwards et al., 2020), or other behaviors related to parental care (Fazekas et al., 2020; Kumari et al., 2022). Overall, the social behavior network could function as a hub for integrating diverse signaling mechanisms that influence context-sensitive singing.

## 2 Role of dopamine in context-sensitive vocalizations

### 2.1 Dopamine production and functional role

First described in macaque monkeys (*Macaca fmcicularis*), dopamine has a key role in denoting the reward prediction error signal that is critical for reinforcement learning (Schultz et al., 1993, 1997). This dopamine-mediated signal often facilitates motor learning (Uehara et al., 2019). Dopamine is primarily synthesized in the ventral tegmental area (VTA; dopamine cell group A10) and substantia nigra (SN; dopamine cell group A9), which together form a continuous region this is herein referred to as VTA. In zebra finches, VTA exhibits context-sensitive activation (Heimovics and Riters, 2005) and neural spiking patterns that are sensitive to self-perceived errors in vocal production (Gadagkar et al., 2016), facilitating subtle adjustments to song from rendition to rendition. The VTA and social behavior network node CG (contains dopamine cell group A11), each innervate three key regions of the songbird vocal control network: HVC (Appeltants et al., 2000; Tanaka et al., 2018), Area X (Lewis et al., 1981; Castelino et al., 2007; Gale et al., 2008), and RA (Appeltants et al., 2002). While these two dopamine-producing regions innervate the same regions for vocal-motor control, the relative densities of these connections are unknown. In canaries, lesioning CG selectively blocks female-directed song while leaving undirected song unaffected (Haakenson et al., 2020; Ben-Tov et al., 2023), suggesting that even if the density of CG projections to the vocal control network are less than that of VTA, the potential contributions to context-sensitive signing is significant. These data suggest that dopamine, either from the VTA or CG, plays a critical role in linking external information (e.g., social cues and self-perceived errors) to learned vocal production.

In the zebra finch, G-protein-coupled dopamine receptors are classified into two major types (Kubikova et al., 2010), excitatory D1-like receptors (D1a, D1b, D1c, and D1d) and inhibitory D2-like receptors (D2, D3, and D4) (Palacios et al., 1988; Neves et al., 2002; Neve et al., 2004; Platania et al., 2012). All cortical regions of the songbird vocal control network express D1-like and D2-like dopamine receptors, while the thalamic node expresses only D1-like dopamine receptor mRNA (Kubikova et al., 2010) (Figure 1A). Dual expression of both D1-like and D2-like receptors suggests that dopamine-mediated signaling in the vocal control network could be additionally dependent on secondary signals from local interneurons or inputs from other brain areas. Blockade of D1-like receptors in HVC reduces the amount of female-directed singing in adult male zebra finches, compared to those who received a saline control (Ben-Tov et al., 2023), suggesting that D1-like receptor activation in HVC may be critical for context-sensitive singing.

A comprehensive analysis of dopamine receptor distribution across the social behavior network has not been performed in zebra finches, although dopamine receptor distributions have been reported in other songbird species. In European starlings (*Sturnus vulgaris*), all social behavior network nodes express D1-like dopamine receptors (Heimovics et al., 2009), while POM expresses both D1-like and D2-like dopamine receptor mRNA (Heimovics et al., 2009; Polzin et al., 2023), and is bidirectionally connected to VTA (Riters and Alger, 2004). Additionally in POM of European starlings, D1 receptor expression is tightly correlated with high singing amounts in the presence of a female (DeVries et al., 2015) (Figure 1B). In zebra finches, localization of tyrosine hydroxylase and absence of dopamine beta-hydroxylase has been reported in multiple social behavior network nodes (Bottjer, 1993; Mello et al., 1998; Alger et al., 2011), indicating dopamine ligand availability. Tyrosine hydroxylase localization in VMH is higher in female zebra finches whose male partners attempted courtship compared to females whose partners did not (Alger et al., 2011), suggesting that dopamine delivery to the social behavior network is sensitive to external cues. Overall, this potential for dopamine-signaling across the social behavior network may facilitate the transmission of socially-informed, rewarding cues to other circuits in the brain, like the vocal control network.

### 2.2 Context-specific activation patterns

VTA activation is positively correlated with female-directed song production and dependent on social context (Heimovics and Riters, 2005). More specifically, VTA neurons that preferentially fire during female-directed singing fire less during undirected song, and vice versa (Yanagihara and Hessler, 2006), indicating that unique populations of neurons within VTA may be responsive to song within a single social context. Additionally, dopamine in Area X is higher in female-directed conditions than in undirected conditions (Sasaki et al., 2006; Ihle et al., 2015). Together these data suggest that neurons within the VTA are sensitive to the social context in which a bird is singing and that context-sensitive signaling from VTA may lead to an upregulation of dopamine in Area X of the vocal control network in female-directed singing contexts. In other songbird species, dopamine signaling is also associated with social singing contexts. In social aviaries, high-singing male and female European starlings express more D1-like (D1b) and D2-like (D2) mRNA in POM of the social behavior network than starlings who sing less (Polzin et al., 2023), increasing sensitivity to dopamine signaling in the social behavior network with increased levels of song. Additionally in starlings, expression of an activity marker in POM and VTA is tightly correlated with singing amount in female-directed contexts, but not in undirected contexts (Heimovics and Riters, 2005). Of course, it is important to note that while these data support a hypothesis that dopamine-mediated activation or inhibition of POM may influence the context-sensitive firing of VTA and ultimate release of dopamine into Area X of the vocal control network, direct influence of the social behavior network on context-sensitive song remains untested.

In one case, a chemical lesion of the locus coeruleus (LoC), a region canonically known for producing a catecholamine norepinephrine positioned immediately next to CG, altered the motor production of song in zebra finches, but chemical lesions within VTA had no effect on song production (Hara et al., 2007). Due to the proximity of LoC and CG, chemical lesioning LoC, likely also lesioned the CG, meaning that the disruption of song may have been due to a disruption of dopamine-synthesizing cells in CG. Similarly, agonizing gamma-aminobutyric acid (GABA_a_), increasing inhibitory signals, in CG resulted in delayed song onset in male canaries (*Serinus canaria*) when presented with a female (Haakenson et al., 2020). The exact interplay between the dopaminergic innervation of the vocal control network by VTA and CG is an unknown, but likely fruitful research question. As outlined in this review, both nodes serve some role in context-sensitive singing.

## 3 Role of oxytocin and vasotocin in context-sensitive song

### 3.1 Oxytocin and vasotocin in vocal modulation

**Figure 2:**
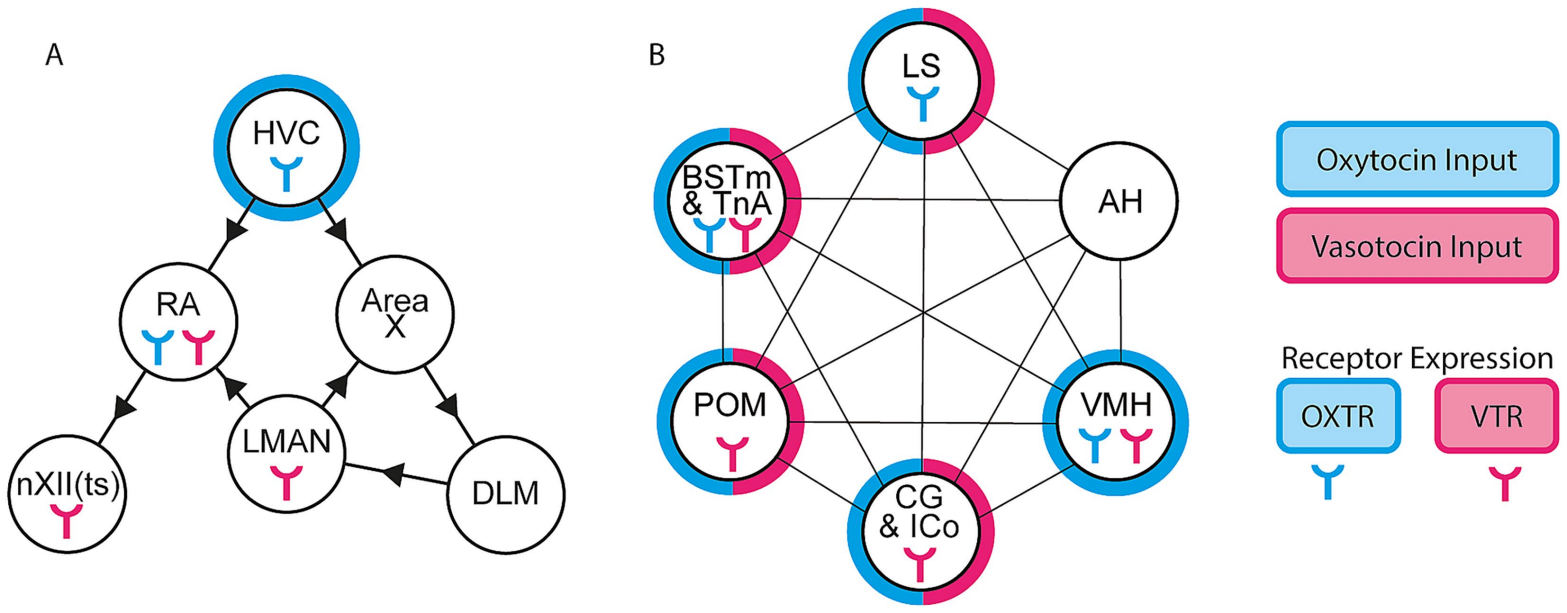
Potential contributions for oxytocin-family signaling to context-sensitive song. Localization of oxytocin receptors (blue icon), vasotocin-family receptors (pink icon), oxytocin ligand (blue outline), and vasotocin ligand (pink outline) in the **(A)** vocal control network and **(B)** social behavior network. Arrowheads between nodes indicate anatomical connectivity, connecting lines without arrowheads indicate reciprocal connections.

Two related molecules, oxytocin and vasotocin, may serve a primary role in facilitating context-sensitive behaviors. Oxytocin and vasotocin are paralogous nonapeptides that bind to a family of G-protein-coupled receptors. There are four orthologous oxytocin-family receptors expressed in avian species: OXTR, VTR1a, VTR1b, and VTR2a (Theofanopoulou et al., 2021). In isolation, activation of the oxytocin receptor or vasotocin receptors by oxytocin or vasotocin, respectively, is typically an excitatory signal (Omura et al., 1999; Bakos et al., 2018). While downstream effects of oxytocin or vasotocin binding to oxytocin-family receptors may be similar, the neural distribution of each receptor subtype is unique and variable across songbird species (Leung et al., 2011; Davis et al., 2022; Marcinkowska et al., 2022). A summary of oxytocin-family receptor expression that has been reported in two songbird species, zebra finch and white-throated sparrows (*Zonotrichia albicollis*), is shown (Figure 2).

Oxytocin and vasotocin are primarily synthesized in the paraventricular nucleus of the hypothalamus (PVN) and the supraoptic nucleus of the hypothalamus (SON) in both songbird and mammalian species, with additional oxytocin synthesis occurring in the BSTM/lateral bed nucleus of the stria terminalis (BSTL) and POM (Montagnese et al., 2015; Grinevich and Neumann, 2021; Haakenson et al., 2022). Many and social brain regions across songbirds receive oxytocin and/or vasotocin ligands. Reported ligand localization in zebra finches and blue tits (*Cyanistes caeruleus*) is collectively summarized (Figure 2). Interestingly, oxytocin ligand distribution has only been reported in one node of the vocal control network, HVC (Haakenson et al., 2022). We wish to highlight the mismatch between reported ligand localization and receptor expression across the vocal control and social behavior networks. This could indicate that nodes with receptor expression but no reported ligand localization could encounter oxytocin-family ligands that are delivered via non-synaptic routes (i.e., diffusion, secretion). Additionally, this could simply be attributed to a lack of data reported. Currently, immunohistochemical tools for detecting oxytocin-family ligands in the songbird are limited. In the future, as GPCR-based sensors for detecting small peptides become more applicable to non-mammalian species (Lee and Kwon, 2022; Ahmed et al., 2023; Qian et al., 2023), a more comprehensive catalogue of oxytocin-family ligand distribution can be compiled in songbirds.

In songbirds, oxytocin and vasotocin have often been studied in relation to non-learned social behaviors. For example, antagonism of oxytocin receptor-mediated signaling in zebra finches reduces the time spent in proximity to conspecifics in large groups (Goodson et al., 2009) and the time spent allopreening (Klatt and Goodson, 2013), suggesting that oxytocin plays a key role in innate affiliative behaviors in this species. Delivery of either oxytocin or vasotocin to juvenile zebra finches decreases or increases, respectively, song similarity to the tutor template (Baran et al., 2017), potentially due to disrupted affiliative behavior. Additionally, adult male zebra finches sang less tutee-directed song to juvenile zebra finches who were treated with an oxytocin antagonist than to saline-treated control juveniles (Pilgeram et al., 2023), suggesting that adults of this species are sensitive to disrupted social systems in conspecifics. Other studies have further investigated the effect vasotocin signaling on modulating learned singing behavior compared to innate behaviors. For example, adult male zebra finches who were treated with a vasotocin antagonist or vasotocin exhibited no change in occurrence of female-directed songs compared to males who received a saline control, but occurrence of aggressive behaviors in these males increased and decreased, respectively, compared to males who received a saline control (Goodson and Adkins-Regan, 1999). While these studies highlight the innate social and learned singing behavioral effects of oxytocin and vasotocin signaling, less is known about the direct mechanisms by which these neuropeptides exert their influence.

### 3.2 Complementary role with dopamine

Mechanisms for social behavior in mammals highlight an integral pathway from PVN to VTA to motor circuits (Hung et al., 2017; Xiao et al., 2017; Borland et al., 2019). Oxytocin-expressing projection neurons in PVN that synapse directly and selectively onto dopaminergic neurons in the VTA are more active following social encounters, increasing firing rates of dopamine neurons in VTA (Hung et al., 2017; Xiao et al., 2017). Additionally, in mammals, disruption of oxytocin signaling in the SN decreases dopamine delivery to a striatal motor region (Sanna et al., 2021). Homologous axonal projections from PVN to VTA in two songbird species, the house finch (*Haemorhous mexicanus*) and white-crowned sparrow (*Zonotrichia leucophrys gambelii*) (Singletary et al., 2006; Ubuka et al., 2012), suggest that a similar mechanism may regulate the context-sensitive switch from more-variable undirected song to directed song in the presence of a potential mate in songbirds. Additionally, in songbirds, oxytocin fibers are present in the VTA (Haakenson et al., 2022), further supporting that a similar mechanism of oxytocin-mediated activation of dopamine neurons occurs in songbirds.

Some suggest that the oxytocinergic POM to dopaminergic VTA pathway in songbirds could function similar to the oxytocinergic PVN to dopaminergic VTA pathway in mammals to regulate context-sensitive behavior in songbirds (Riters and Alger, 2004; Theofanopoulou et al., 2017). However, it should be recognized that in songbirds, POM is bidirectionally connected to the PVN. Activation of PVN and POM is correlated during female-directed, but not undirected, singing contexts (Anderson et al., 2023). This dual activation of POM and PVN suggests a functional relationship between POM and PVN in regulating context-specific singing behavior in songbirds. These findings connect POM to the well-documented PVN-to-VTA pathway, which mediates context-sensitive behaviors in mammalian species.

## 4 Connectivity between vocal control and social behavior networks

### 4.1 Functional connectivity

Correlational activity between distinct brain regions, i.e., functional connectivity evidence, links the songbird social behavior network to the vocal control network in context-sensitive singing. Distinct patterns of neural activity-dependent gene expression across the social behavior network in adult male zebra finches is correlated with context-specific singing (Anderson et al., 2023). Furthermore, correlated activity between node pairs within this social network is dependent on social context (Anderson et al., 2023). For example, after a period of undirected singing, activation of POM is positively correlated with that of CG, but after female-directed singing POM activation is no longer correlated with CG, but instead correlates with activity in the BSTM, LS, AH, and VMH (Anderson et al., 2023). In another songbird species, European starlings, unique patterns of neural activity-dependent gene expression further demonstrate the context-specific activity of the social behavior network. In breeding-contexts, neural activity-dependent gene expression in LS is negatively correlated with singing, while expression of the same gene in VMH increases with singing (Heimovics and Riters, 2007). Together, these finding demonstrate a functional relevance for the social behavior network in regulating context-sensitive song, likely through dynamic reorganization of its activity across different social contexts. However, it is important to note that context-sensitive expression of an activity-dependent gene in various nodes of the social behavior after song production, is simply correlational evidence that activity in the social behavior network is involved in context-sensitive singing.

### 4.2 Anatomical pathways

**Figure 3:**
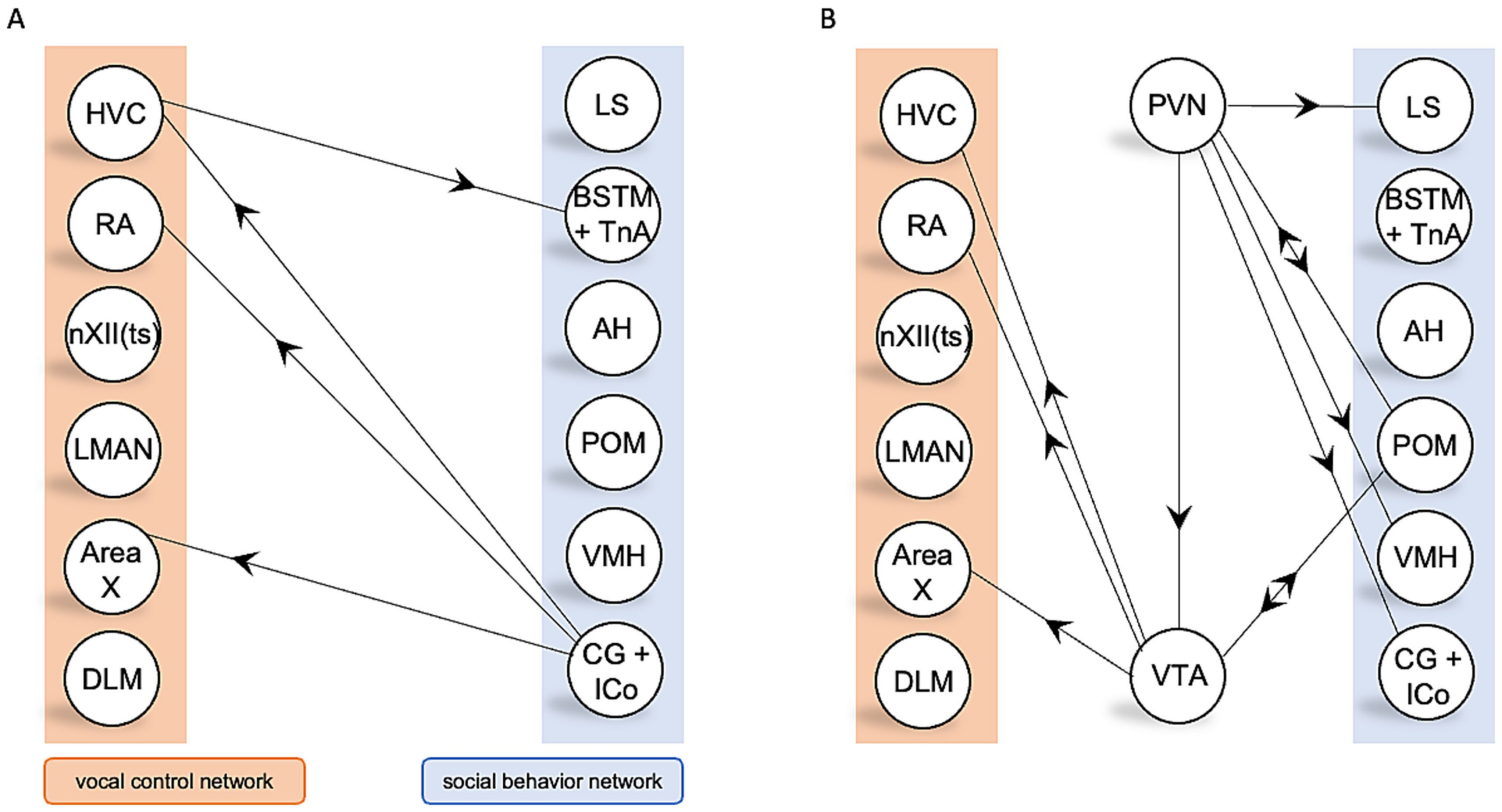
PVN and VTA serve as an anatomical bridge between the songbird vocal control and social behavior networks. **(A)** Direct anatomical connections between the vocal control network and the social behavior network. **(B)** Indirect anatomical connectivity between the social behavior network and the vocal control network, utilizing PVN and/or VTA as liaison nodes. All connections shown have been reported in at least one songbird species.

Few studies have reported direct anatomical connectivity between the social behavior network and the vocal control network. Notably, CG of the social behavior network, which produces dopamine, innervates HVC, Area X, and RA (Appeltants et al., 2000, 2002; Castelino et al., 2007; Tanaka et al., 2018) (Figure 3A). Additionally, HVC innervates TnA, potentially allowing for vocal-motor feedback to the social behavior network (Cheng et al., 1999) (Figure 3A). However, this limited number of studies does not necessarily imply a lack of connectivity between these networks, this could reflect of songbird research to the vocal control network. As of 2024, the only nodes of the songbird brain that have been directly injected with an axonal tract tracer in over 10 different studies are HVC, Area X, LMAN, RA, and the caudolateral nidopallium (NCL) (Savoy et al., 2024). The vast majority songbird brain regions have only been the direct target of a tracer study one or zero times (Savoy et al., 2024).

Considering two- and three-synapse anatomical connections, rather than direct ones, the social behavior network may communicate with the vocal control network using interactions between oxytocin-producing PVN and dopamine-producing VTA. Many nodes of the social behavior network receive axonal input from PVN in songbirds (Figure 3B), suggesting a role for PVN as a hub for oxytocin-mediated modulation of the social behavior network. Further, in zebra finches, POM is reciprocally connected to PVN (Riters and Alger, 2004), potentially creating a feedback loop between the social behavior network and PVN.

In non-songbird avians and mammals, PVN and VTA are bidirectionally connected, whereas in songbirds, only a PVN-to-VTA connection has been reported (Singletary et al., 2006; Ubuka et al., 2012). Additionally, in two non-songbird avian species, the domestic chicken (*Gallus gallus*) and the domestic mallard (*Anas platyrhynchos domesticus*), LS innervates PVN (Korf, 1984; Montagnese et al., 2004). The lack of reported songbird connections could be due to a bias to investigate the specialized song system, rather than generalized exploratory characterization studies of the rest of the brain. Nonetheless, these findings suggest that PVN may act as an anatomical bridge between the social behavior network and vocal control network.

## 5 Discussion

### 5.1 Proposed model of dopamine-oxytocin interactions in context-sensitive singing

**Figure 4:**
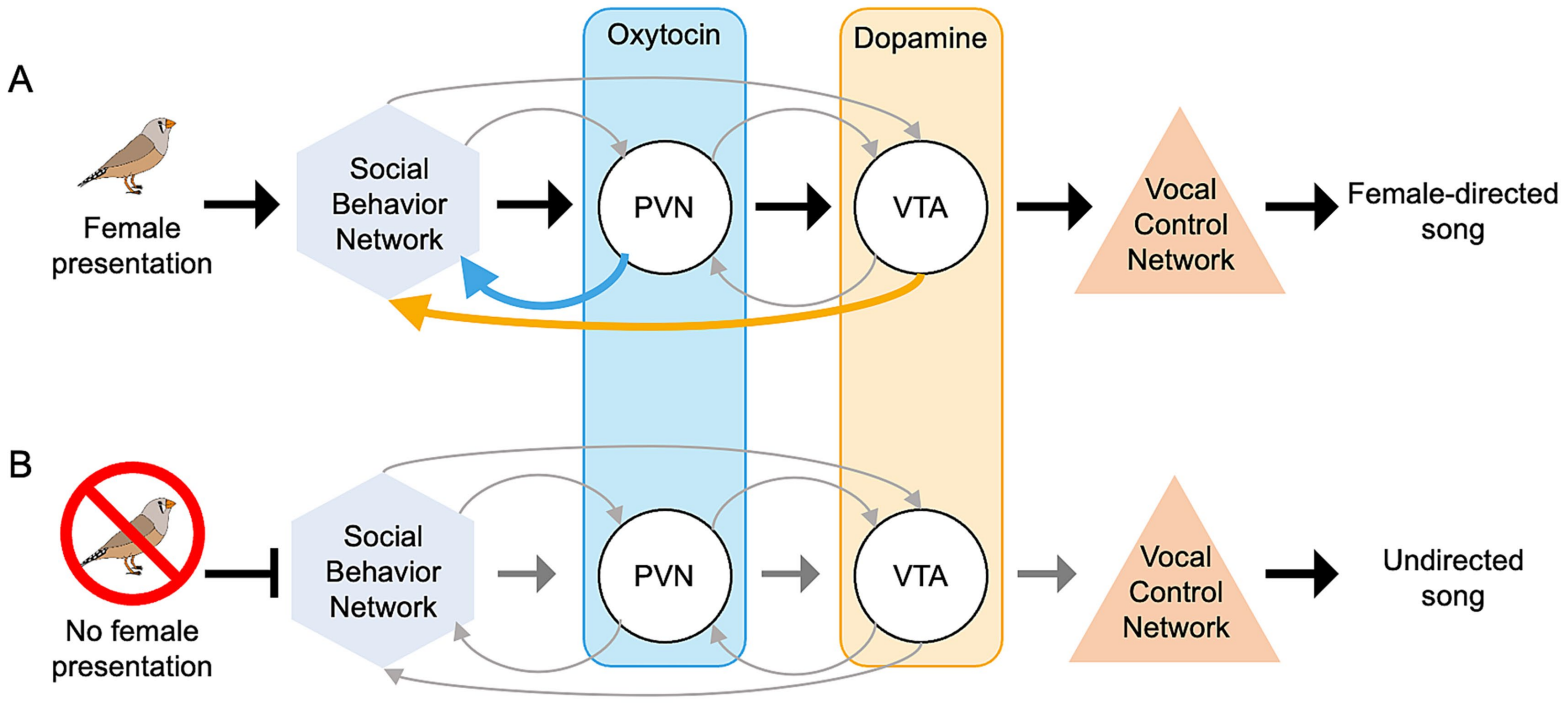
Proposed model for context-sensitive singing. Context-sensitive modifications to song in the adult male zebra finch are potentially mediated by a synaptic pathway moving through the social behavior network, PVN, VTA, and ultimately the vocal control network. **(A)** An increase in overall social behavior network activation, potentially caused by social oxytocin and rewarding dopamine input signals, triggers a context-specific signature of activation in the vocal control network, allowing for female-directed singing. **(B)** A decrease in overall social behavior network activity, potentially caused by baseline or reduction in baseline oxytocin and dopamine input to the network, could facilitate undirected singing.

Considering molecular, anatomical, and functional brain data in songbirds, we hypothesize that the POM may serve as the output nucleus of the social behavior network, influencing oxytocin-mediated dopamine delivery to the vocal control network, in a context-sensitive manner (Figure 4). These neuromodulatory signals may also engage feedback mechanisms enabling dynamic self-regulation within the pathway to respond to changing social contexts in real time. In highly stimulating social settings (i.e., immediately following the presentation of a potential mate), activation of POM is tightly correlated with activation of both PVN and VTA, compared to periods of undirected singing where POM activity is correlated with activation of CG. Potentially, in social environments, POM activity is released from CG oversight to go on to activate oxytocin-producing neurons in PVN. In turn, oxytocin PVN neurons synapse onto dopamine neurons in the VTA that express oxytocin receptors, curating a signaling relationship that is essential for the reinforcement of social behaviors (Borland et al., 2018). This cascade resulting in VTA activation could be responsible for the increase in dopamine delivery to the vocal control network, as has been reported during periods of female-directed singing. The anatomical connections to facilitate the proposed pathway have each been reported in songbird species (Savoy et al., 2024), and functional data reported in many songbird species seems to support this hypothesis. Reciprocal connections among many of the social behavior network nodes, PVN, and VTA suggests the potential for self-regulation within this pathway.

### 5.2 Potential conservation of proposed model across vertebrates

By drawing parallels with mammalian systems, the proposed model for zebra finches provides an opportunity to uncover universal principles governing social influence on motor behaviors, with specific adaptations for vocal learning. Context-sensitive behavior, which is, in essence, the adjustment of behavioral responses to stimuli based on environmental and social contexts, is adaptive for many animal species. Each vocal learning species can manipulate their vocalizations, albeit speech or song, in some capacity. These species fall into two major groups: mammalian (i.e., humans, elephants, whales, dolphins, seals, bats) and avian (i.e., songbirds, parrots, hummingbirds). Like humans, bottlenose dolphins (*Tursiops truncatus*) and African elephants (*Loxodonta africana*) use specific vocalizations or alter their vocalizations when addressing unique members of their community (Sayigh et al., 2023; Pardo et al., 2024). Additionally, harbor seals (*Phoca vitulina*) can also learn new vocalizations and use them in specific contexts (Ralls et al., 1985; Duengen et al., 2024). Remarkably, a few species can mimic human speech in socially rewarding contexts, despite biological differences. The ability of vocal-learning species to modify their vocalizations based on social and environmental contexts raises intriguing questions about the neural mechanisms underlying social influences on vocal modifications.

**Figure 5:**
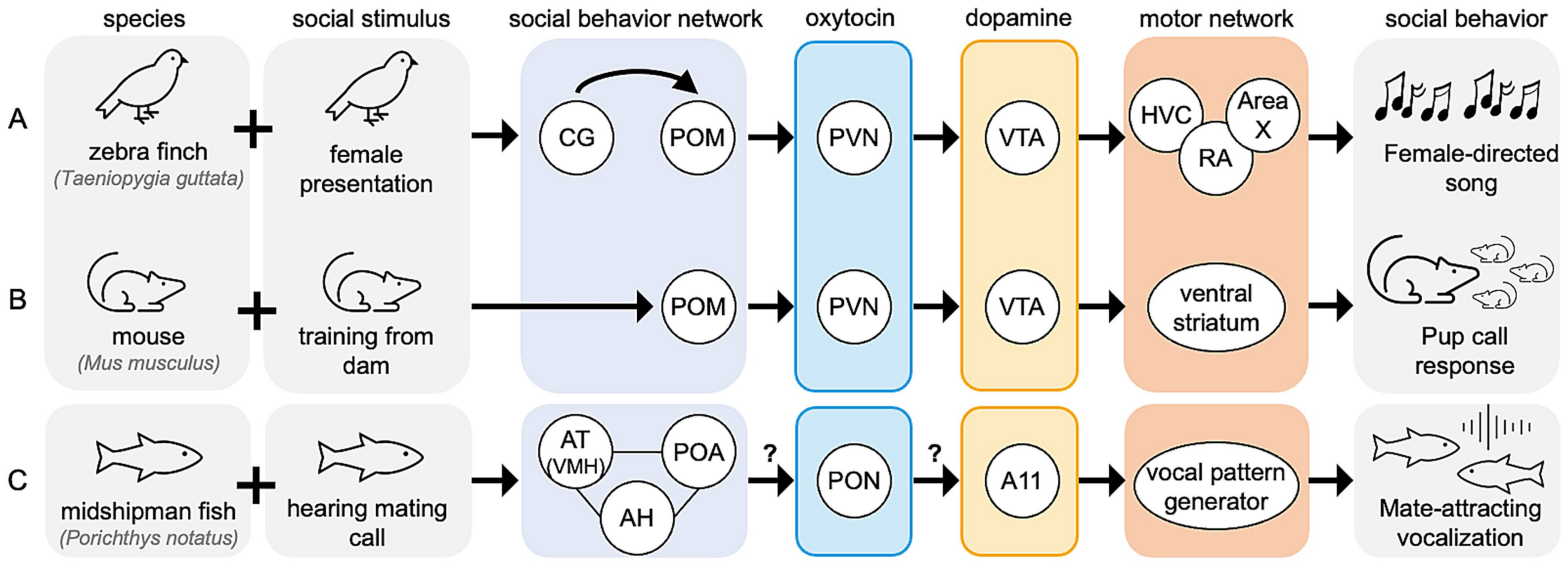
Expansion of proposed model across vertebrates. Hypothesized node-level mechanisms for social context-sensitive motor behaviors are shown for **(A)** zebra finch, **(B)** mouse, and **(C)** midshipman fish. All nodes shown have reported functional relevance for context-sensitive motor behaviors in their respective species. Question marks over arrows represent unreported functional connections.

While our proposed mechanism is specific to context-sensitive singing behavior in songbirds, similar neural mechanisms may facilitate context-sensitive behavior in non-vocal-learning species (Figure 5). Homologous regions to the songbird social behavior network, PVN, and VTA are found across vertebrates. In species without direct analogs to the songbird vocal control network, VTA may innervate a non-specified motor network, as it does in mouse models to facilitate social-directed motor behaviors (Hung et al., 2017).

In mice, experienced mothers respond quickly to distress calls made by mouse pups outside of the nest, while naïve females may ignore the pups completely. Therefore, the motor response to mouse pup calls is a learned, or experience-driven behavior. Naïve females can learn these pup care behaviors from experienced to naïve females, in a social process mediated by oxytocin-expressing PVN neurons (Carcea et al., 2021). In adult female mice, activation of oxytocin receptor-expressing neurons in POM is positively correlated with pup exposure, and oxytocin expression is higher the PVN and SON of high pup exposure groups compared to low pup exposure groups (Okabe et al., 2017). Additionally, oxytocin-expressing PVN neurons are more active during mouse pup care behaviors than during infanticide, and optogenetic activation of these neurons reduced infanticide behaviors (Li et al., 2024), linking oxytocin signaling to the learning of mouse pup care behavior. Activation of VTA dopamine neurons is also positively correlated with mouse pup retrieval; inhibiting these neurons increases retrieval delay compared to response times in uninhibited mice (Xie et al., 2023). Similarly, endogenous dopamine release in the ventral striatum, a region influenced by VTA activity increases in maternal rats (Hansen et al., 1993). Given that POM synthesizes oxytocin, it is unclear whether the activation of oxytocin receptor neurons in POM is self-generated or mediated by another nucleus. However, PVN innervates VTA in rodents, suggesting this oxytocinergic signaling in PVN may directly trigger dopamine release in the ventral striatum via VTA, facilitating context-sensitive motor behavior. Furthermore, oxytocin-mediated dopamine signaling in vertebrate species may play a conserved role in social behaviors, as recently reviewed (Rappeneau and Díaz, 2024). Of course, while the adult female mice do not learn vocal responses to pup calls, they exhibit a learned motor behavior that is socially driven. In this way, this response is the mouse is context-sensitive and may be under control of a neural mechanism similar to that in the songbird (Figure 5B).

In another vertebrate species, the midshipman fish (*Porichthys notatus*), multiple nodes of the social behavior network are responsive to mating-specific calls. The fish homologue of VMH (anterior tuberal nucleus of the hypothalamus; AT) selectively responds to conspecific mating calls and not calls from other fish species or ambient noise (Mohr et al., 2018). Additionally, the continuous homologue of AH and POM, express transcriptional profiles that are correlated with social vocalizations, regardless of likelihood of eventual mating success (Tripp et al., 2018). Additionally, in midshipman fish, many nodes of the social behavior network express oxytocin receptors that are activated in males performing mating-related vocalizations (Schuppe et al., 2022). While oxytocin-mediated dopamine signaling has not been reported in the midshipman fish in reference to context-sensitive vocalizations, this type of dopamine regulation has been reported in another teleost species, the catfish (*Claria batrachus*) (Singh et al., 2016), here this oxytocin originates in the preotic nucleus (PON). Dopamine signaling in the midshipman fish brain is increased in response to the perception of social vocalizations. Dopamine cell group A11 neurons, are selectively activated in the midshipman fish after exposure to a mating hum call compared to ambient noise (Ghahramani et al., 2018), and spike trains of some A11 neurons are tightly correlated with the onset of vocalizations (Kittelberger et al., 2006). In songbirds A11 is physically within CG/ICo, but in teleost fish these dopaminergic cells may be functionally more similar to VTA (Petersen et al., 2013). Further, dopamine injections into the fish A11 reduce the probability of vocalizations, an effect that is blocked by co-injection with a non-selective D1/D2 receptor antagonist (Allen et al., 2023). This may reflect a feedback mechanism, where locally applied dopamine acts via D2-like receptors to inhibit dopaminergic activity in A11, perhaps to refine the timing or duration of vocalizations. In summary, these teleost fish exhibit many of the characteristic phenotypes predicted by our proposed model for context-sensitive vocalizations (Figure 5C), despite being evolutionarily distant from songbirds.

Our model, derived primarily from existing literature in songbirds, suggests that in response to social stimuli the social behavior network oversees oxytocin-mediated activation of dopaminergic cells such as those in A11/CG/ICo, as well as the VTA, to facilitate context-sensitive motor behaviors. The species reviewed here span millions of years of evolutionary diversity, yet in each we find evidence for the conservation of the social behavior network facilitating oxytocin-mediated activation of dopaminergic cells, which in turn impact various the circuits for motor-control. This conservation of functional, anatomical, and effective connectivity across vertebrates highlights the potential of our model as a shared neural mechanism underlying context-informed motor behaviors.

### 5.3 Potential for future studies

To advance our understanding of the brain mechanisms underlying context-sensitive behavior, future studies could prioritize investigating cross-network neurotransmitter interactions rather than focusing solely on isolated molecules or pathways. Neural processes rarely act in isolation. While reductionist approaches are invaluable for generating interpretable data from complex systems, focusing solely on isolated processes may overlook the broader interactions and integrations that underlie context-sensitive behaviors. We emphasize a previous call for more basic investigations of the social behavior network (Kelly, 2022). While the social behavior network was hypothesized nearly 20 years ago to be conserved across all vertebrate species, comprehensive anatomical evidence for its interconnectivity remains incomplete in many of the model organisms reviewed here.

**Figure 6:**
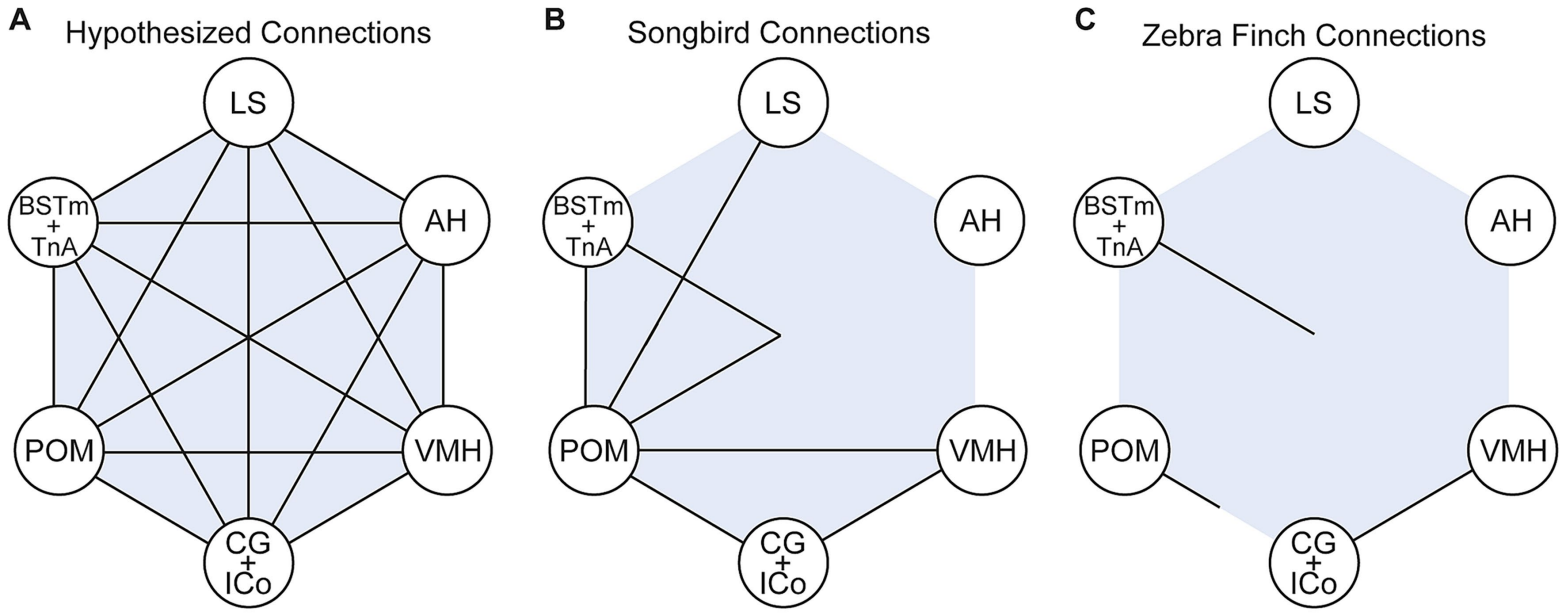
Verified interconnectivity of the social behavior network. **(A)** Hypothesized fully interconnected social behavior network. **(B)** Anatomical connections between nodes of the social behavior network that have been verified via tract tracing studies in songbird species. **(C)** Anatomical connections between nodes of the social behavior network that have been verified via tract tracing studies in zebra finches.

In songbirds, strong functional evidence supports the role of the social behavior network in context-sensitive singing, as demonstrated in species such as zebra finches and European starlings. However, anatomical connectivity data for the social behavior network in any songbird species is far from complete (Figure 6A). Of the 30 expected anatomical connections between all six nodes of the social behavior network, only 12 have been reported in songbird species (Figure 6B), with even fewer connections confirmed in a single species, the zebra finch (Figure 6C). Many of these connections have been inferred from studies in non-songbird avian species, such as quail. Resources like the recently published songbird connectome (Savoy et al., 2024), which maps all reported anatomical connections in the songbird brain, provides a valuable tool to identify understudied social behavior network nodes and connections. Characterizing the social behavior network within a single species would provide critical evidence for its conserved structure and function. Future studies could use immunohistochemical or *in situ* hybridization techniques to investigate whether cells within social behavior network nodes are responsive to dopamine and oxytocin signaling, either separately or in combination. Multi-label histology studies could determine whether these cells express dopamine and oxytocin-family receptors, highlighting their potential for co-modulation. Such work could inform functional studies testing the effects of disrupting specific nodes or signaling pathways on context-sensitive behaviors, including vocalizations.

In addition to the social behavior network, recent studies emphasize the importance of integrating other specialized networks, such as the auditory system, into models of context-sensitive song behavior in birds. For example, inhibition of the auditory system using GABA receptor agonist diminishes sound-triggered activation of the lateral VMH of the social behavior network (Spool et al., 2024). This finding suggests that auditory signals prime the social behavior network to anticipate social interactions, reinforcing the need for studies exploring the integration of defined networks into models of context-sensitive behavior. Yet another promising avenue is the role of the caudolateral nidopallium (NCL), an integrative brain region analogous to the mammalian prefrontal cortex that is involved in planning context-sensitive vocalizations in a songbird crow species (*Corvus corone corone*) (Brecht et al., 2023). The NCL may coordinate with the social behavior network to modulate vocal control network activity and context-sensitive behaviors. Investigating its contributions to decision-making, social context processing, and vocal-control could provide insight into how learned behaviors are flexibly adapted to changing contexts.

Finally, female zebra finches do not sing, but they exhibit context-sensitive responses such as strong behavioral preferences for the song of their pair-bonded mate (Woolley and Doupe, 2008). These preferences are maintained by D2-like dopamine receptors (Day et al., 2019) expressed in POM of the social behavior network. Female zebra finches may establish song preferences through a mechanism similar to the model we propose here for supporting context-sensitive of learned vocalizations in males. Investigating the anatomical connectivity between the social behavior network and the female vocal control network, as well as the functional contributions of these interactions, may reveal pathways that enable subtle vocal adjustments indicating song preferences, such as reactions to male song.

In summary, further basic research into the anatomical connectivity and cellular composition of the social behavior network within a single species, combined with functional studies to identify the relevant targets and effective connectivity studies targeting specific nodes is essential to understanding its role in context-sensitive behaviors. By integrating additional networks and neurotransmitters, future research may build a more comprehensive model of the neural mechanisms underlying context-sensitive vocal and social behavior.
